# Temporal Analysis of COVID-19 Epidemiological Indicators in a Low-Income Brazilian Context: A Retrospective Analysis in Paraiba State

**DOI:** 10.3390/v15102016

**Published:** 2023-09-28

**Authors:** Fabiola Ferreira da Silva, Luiz Carlos de Abreu, Blanca Elena Guerrero Daboin, Tassiane Cristina Morais, Matheus Paiva Emidio Cavalcanti, Italla Maria Pinheiro Bezerra, Célia Guarnieri da Silva, Fernando Augusto Marinho dos Santos Figueira, Viviane Valeria de Caldas Guedes, Andres Ricardo Perez Riera

**Affiliations:** 1 Laboratory of Studies Design and Scientific Writing, Postgraduate Division, University Center FMABC, Santo André 09060-870, SP, Brazil; fabiolaferreira_silva01@hotmail.com (F.F.d.S.); luizcarlos@usp.br (L.C.d.A.); blanca.daboin@fmabc.br (B.E.G.D.); celia.guarnieris@gmail.com (C.G.d.S.); andres.riera@gmail.com (A.R.P.R.); 2 Department of Integrated Health Education, Federal University of Espirito Santo, Vitoria 29075-910, ES, Brazil; 3 School of Medicine, University of Limerick, V94 T9PX Limerick, Ireland; 4 Post-Graduate Program in Medical Sciences, Faculty of Medicine of the University of São Paulo, São Paulo 01246-903, SP, Brazil; mpaivaemidio@gmail.com (M.P.E.C.); fernando.figueira@usp.br (F.A.M.d.S.F.); 5 School of Sciences of Santa Casa de Misericórdia de Vitória (EMESCAM), Vitoria 29045-402, ES, Brazil; italla.bezerra@emescam.br; 6 Collective Health, Catholic University of Santos, UNISANTOS, Santos 11015-001, SP, Brazil; vivinhaguedes@hotmail.com

**Keywords:** COVID-19, epidemiology, incidence, mortality, trends, Paraiba, Brazil

## Abstract

Northeast Brazil is a region with great international tourist potential. Among the states that make up this region, Paraíba stands out due to the presence of vulnerable groups and factors that contribute to adverse outcomes of COVID-19. Therefore, the aim of this study was to analyze the epidemiological data on the incidence, mortality, and case fatality of COVID-19 in Paraíba. An ecological, population-based study was performed, with data extracted from the Brazilian Ministry of Health database. All cases and deaths from COVID-19 from March 2020 to December 2022 were included. The time series was built by applying the Prais–Winsten regression model, and the daily percent change was calculated to analyze the trends. The highest case fatality of the entire period was in April 2020 (7.8%), but in March 2021, the state broke the dismal record of 1248 deaths and the highest mortality rate (30.5 deaths per 100,000 inhabitants). Stationary mortality and case fatality were better in 2022; however, in February 2022, the mortality rate was at levels similar to the same month of the previous year. These results illustrate that COVID-19 is evolving and needs to be constantly monitored.

## 1. Introduction

COVID-19 represents the most significant global health collapse of the century and one of the most critical threats the world has faced since the Second World War [1]. Coping measures to mitigate the transmission of the virus and control the spread of the pandemic had worldwide repercussions on the economic, social, political, psychological, and environmental levels and affected billions of individuals. In addition, COVID-19 also led to changes in lifestyle, work activities, and social interactions, with the potential for more significant harm to populations in vulnerable situations [2,3]. Globally, until the beginning of March 2023, the European continent recorded the highest number of confirmed disease cases. However, it was the countries of the Americas that broke sad world records in deaths, particularly Brazil, which occupies second place in the world ranking of fatal cases of COVID-19 [4], a fact that confirmed the country and Latin America to be the epicenter of the disease in critical moments during the pandemic [5].

The Brazilian Unified Health System (SUS) provides free care to the population. The country has 5570 municipalities occupying a territorial area of 8,510,345,540 km² [6] and faces numerous racial and sociodemographic disparities that directly affect the outcomes and injuries arising from COVID-19 [7]. This fact corroborates the different scenarios presented in the incidence, mortality, and case fatality of COVID-19 described in the scientific literature by numerous studies in different regions of the country [8,9,10,11].

The southeast region, where large internationally known metropolises such as São Paulo and Rio de Janeiro are located, was the most affected region [12]. However, few studies address the behavior of COVID-19 in the Northeast, which has a substantial portion of municipalities with high social vulnerability and a low Human Development Index (HDI) [13]. Cities with a low quality of life index and regions with more significant deficits in vaccination coverage, due to limited resource availability and low socioeconomic status [14]. Nevertheless, the Northeast has become a vital tourist reception territory [15]. National and international tourists visit the region searching for sun and sea, luxury, eco-tourism and rural tourism, art, culture, and gastronomy. This activity represents the sustainability and economic advancement of the Brazilian northeast, especially after the difficulties faced during the pandemic [16]. 

In the northeast region, Paraíba stands out; 68% of its municipalities have a low human development Index (HDI). Vulnerable groups live in rural areas or the peripheries of cities lacking essential sanitary services. There are almost 25,000 indigenous people [6] and 39 communities of racial-ethnic groups of Black ancestry associated with resistance and historical oppression. Further, approximately 1500 gypsies live in Paraíba, and more than 95% of them are located in remote zones [17]. 

In crises, special groups must receive constant attention, and clear and precise policies and programs must be developed to support them [2]. Therefore, it is essential to monitor the behavior of COVID-19 in these regions so they can develop effective coping strategies that consider the health of the community and its sociocultural needs, which, when implemented, can equalize access to health care and reduce inequalities caused by health determinants that contributed to adverse outcomes during the pandemic [7,18]. Therefore, this study retrospectively analyzes the incidence, mortality, and case fatality of COVID-19 in Paraíba and the dynamics of those indicators from March 2020 to December 2022.

## 2. Materials and Methods

### 2.1. Study Design and Population

We conducted an ecological, population-based study with data extracted from the Brazilian Ministry of Health databases, which are official sources and do not show personal identification of the examined population [12].

The database was updated on 23 January 2023. All cases and deaths from COVID-19 from March 2020 to December 2022 in Paraíba, northeastern Brazil, were included in the study.

Among Brazilian states, Paraiba ranks fourth from the bottom in terms of the Human Development Index (HDI), which measures its population’s overall well-being and living standards. Specifically, its HDI is 0.658. Out of the 223 municipalities in Paraiba [6], a significant majority, approximately 68%, have a relatively low HDI (Table 1).

The healthcare system in Paraiba is organized into 12 administrative units, each overseen by the State Health Department. These units are responsible for managing and coordinating healthcare services across the state.

To break it down further, let us consider the different regions within Paraiba. For instance, Region 1 is home to 40% of the state’s total population. Within this region, a substantial 76% of its 25 municipalities have a lower HDI, indicating that a significant portion of the population in Region 1 faces challenges related to their overall well-being and living conditions [6].

### 2.2. Inclusion and Exclusion Criteria and Data Collection

In our study, we included all cases and deaths of individuals who had been diagnosed with COVID-19 through laboratory tests and had sought medical treatment. We categorized these cases based on the International Classification of Diseases, 10th edition (ICD-10), specifically as either “U07.1 COVID-19, identified virus” or “U07.2 COVID-19, virus not identified”. Cases and deaths were classified according to the notification date [12].

We organized and tabulated this data in Excel to create the figures and tables in our research. To ensure the accuracy of our data, two different researchers independently collected and verified the information. The principal author of this study retains all the raw data in case it is needed for further examination or verification in the future refers to the source or reference that provides details on how we conducted this data collection and organization process.

### 2.3. Data Analysis

We calculated the incidence and mortality rates by considering 100,000 inhabitants. We assessed how many new cases or deaths occurred for every 100,000 people per year analyzed. Additionally, we calculated the case fatality, expressed as a percentage, to understand the proportion of people who died among those diagnosed with COVID-19. These calculations allow us to describe the impact of COVID-19 on a standardized scale, making it easier to compare across different populations and periods. We used the mathematical Equations (1)–(3) to perform these calculations, as follows:

(1) $$Incidence=\frac{number\;of\;cases}{Population}\times 100.000$$

(2) $$Mortality=\frac{number\;of\;deaths}{Population}\times 100.000$$

(3) $$Case-fatality=\frac{number\;of\;deaths}{\;number\;of\;cases}\times 100$$

In the calculation of incidence and mortality, the population was taken from “Population Projection of the Federation Units by sex and age groups was used: 2000–2030” for 2020 (4,097,859 inhabitants), 2021 (4,119,993 inhabitants), and 2022 (4,141,161 inhabitants) [19]. In our study, we wanted to examine how COVID-19 indicators changed over time. To do this, we divided the study period into three segments: 2020 (from March to December), 2021 (covering January to December), and 2022 (spanning January to December). This allowed us to see how these events evolved throughout these specific timeframes.

To conduct our trend analysis in a rigorous and established manner, we used time series analysis as described by Antunes and Cardoso. Time series analysis is a method used to organize and understand quantitative data collected over a period of time. It is particularly valuable because it enables us to predict past and future values based on statistical calculations, primarily through linear regression [20]. 

We constructed the time series for our analysis using the Prais–Winsten regression model, commonly used in epidemiological studies. This model was employed to consider the influence of first-order autocorrelation when analyzing the time series data. The data modeling process included transforming rates (dependent variable = Y value) into a base 10 logarithmic function, and the results of the logarithmic rates (*β*) from the Prais–Winsten regression allowed for estimating the DPC for the studied region, along with their respective 95% confidence intervals. The values for probability (p) and daily percent change (DPC), considering a 95% level (CI95%) of significance, were calculated using Equations (4)–(6), where *β* is the angular coefficient from the linear regression, the indexes *ul* mean the upper limit, and *ll* is the lower limit of the confidence level [20,21].

(4) $$DPC=\left(10^{\beta }-1\right)\times 100\%$$

(5) $$({CI95\%)}_{ul}=(10^{\beta_{max}}-1)\times 100\%$$

(6) $$({CI95\%)}_{ll}=(10^{\beta_{min}}-1)\times 100\%$$

Essentially, it helps us understand how past data points relate to current ones, which is crucial in assessing and categorizing incidence, mortality, and case fatality trends as either increasing (*p*-value < 0.05 and positive beta), decreasing (*p* < 0.05 and negative beta), or remaining stable (*p*-value ≥ 0.05) [20]. When we say a trend is “flat,” it means there is no significant change in the data over time. 

Statistical analyses were performed using the STATA 14.0 software (College Station, TX, USA, 2013).

## 3. Results

**Figure 1:**
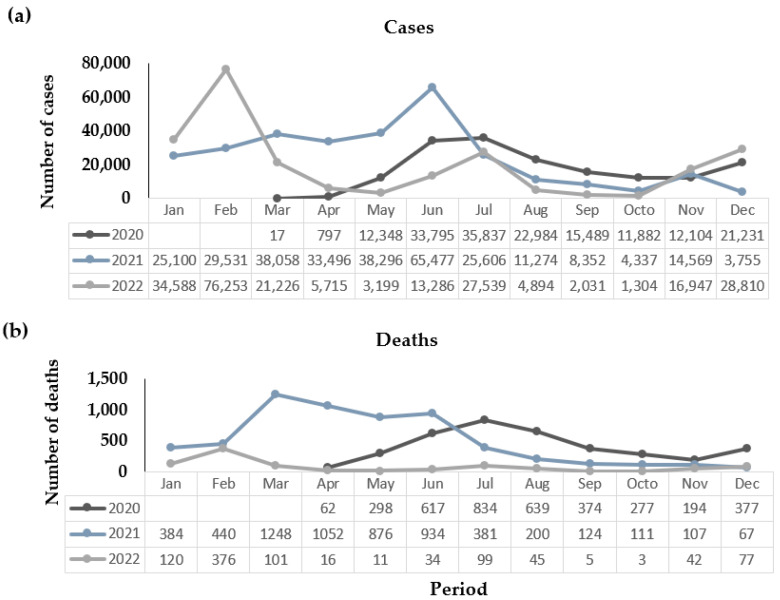
Distribution of cases (**a**) deaths (**b**) by COVID-19 in Paraíba, northeastern Brazil, from March 2020 to December 2022.

In Figure 1, we can observe that the first cases of COVID-19 in Paraiba were documented in March 2020, with a total of 17 reported cases. Just one month later, in April 2020, there was a significant spike in cases, reaching a total of 797, and sadly, 62 (8%) of these cases resulted in fatalities.

Following this initial surge, the number of COVID-19 cases and associated deaths in Paraiba continued to rise exponentially. Fast forward three years to the end of 2022, and the state had recorded a staggering 700,127 cases and 10,525 deaths attributed to the virus. The year 2021 marked the highest point regarding both cases and fatalities. Compared to the previous year, there was a nearly 90% increase in cases, accounting for 297,851 cases. The number of deaths also rose by approximately 54%, representing 5924 more fatalities.

Interestingly, March 2021 saw the highest number of fatalities during the entire period, with 1248 recorded deaths. However, it is noteworthy that the highest volume of cases was reported in February 2022, nearly two years after the pandemic’s onset, with a total of 76,253 cases documented that month.

**Figure 2:**
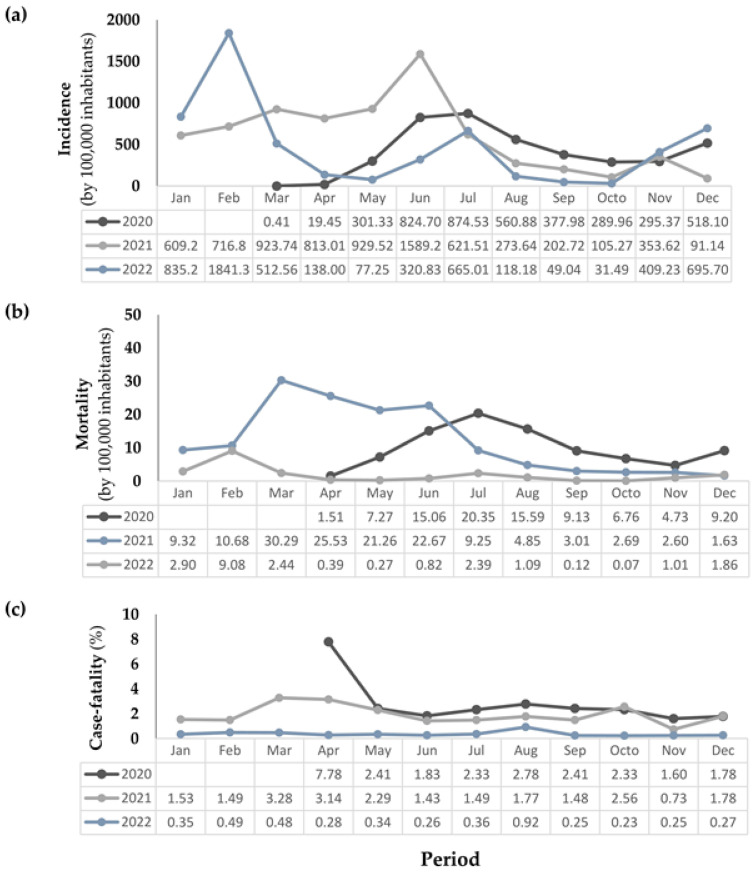
Distribution of incidence (**a**), mortality (**b**), and case fatality (**c**) from COVID-19 in Paraíba, northeastern Brazil, from March 2020 to December 2022.

The epidemiological statistics for the disease exhibited fluctuations throughout the period under review. Figure 2a indicates that the highest incidence rates were observed in June 2021, with 1589.2 cases per 100,000 inhabitants. Eight months later, in February 2022, the peak of incidence for the entire analyzed period occurred, reaching 1841.3 cases per 100,000 inhabitants.

At the beginning of the pandemic, there was a notably high case fatality rate of 7.78% in April 2020, as depicted in Figure 2c. However, in 2021, there were significant spikes in mortality, particularly in March and April, with 30.29 and 25.53 deaths per 100,000 inhabitants, respectively, as shown in Figure 2b.

Our research shows that the disease’s patterns changed over the three years. In 2020, the incidence and mortality increased by 1.72% and 0.42% per day, respectively, while the percentage of case fatalities reduced by 0. 40% per day. However, despite these negative trends observed for incidence and mortality with statistical significance (*p* < 0.05), 2021 saw an upward trend, with an increase of 0.15% per day in case fatality. 

In contrast, a better picture was revealed in 2022; the mortality and case fatality remained stable. Table 2 provides more detailed statistical information, including the Prais–Winsten regression estimates and the DPC for COVID-19 incidence, mortality, and case fatality. 

## 4. Discussion

Our findings indicate that pandemic waves presented distinct situations over the three years in Paraiba. The first wave was marked by a high virus circulation between March and December 2020 [22]. The second, from February to July 2021, marked the emergence of several variants, and the third, from December 2021 to December 2022, was characterized by the presence of the Omicron variant. Since the beginning of the pandemic, replacements of the variants have been observed in the period analyzed [22,23]. The study by Naveca et al. [24] suggests that these replacements result from the heterogeneity in social distancing initiatives and the emergence of more contagious variants.

The disease caused by the SARS-CoV-2 virus spread to all state municipalities, and at the end of 2022, the case fatality and mortality from COVID-19 showed a stationary trend. However, as the pandemic constantly evolves and epidemiological data fluctuates, monitoring its mortality, case fatality, and incidence trends is still necessary. We can see distinct features for each year when examining how COVID-19 epidemiological indicators changed in Paraiba in 2020, 2021, and 2022. Because of these differences, we will study each year separately to understand its unique characteristics better.

**Figure 3:**
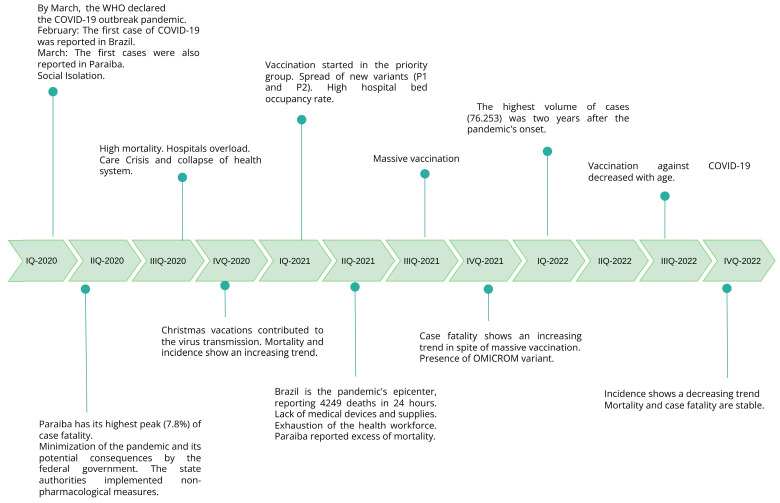
Timeline of significant events over the three years of the COVID-19 pandemic in Paraiba. Q = Quarter.

Figure 3 visually illustrates important events that occurred over the timeline of the three years of the COVID-19 pandemic in Paraiba and how these events affected the trends in reported COVID-19 cases and fatalities during that time.

### 4.1. 2020: Anxiety, Fear, and Misinformation

The first year of the pandemic in Brazil was marked by controversies associated with the response of the national government [25]. Implementing social distancing actions and other non-pharmacological measures depended on each state’s government [26]. The position of minimizing the pandemic and its potential consequences on the federal government impacted the spread of the virus.

In December 2020, Paraiba reported an incidence higher than that of the northeast region (2931.70 cases per 100,000 inhabitants), also above that reported at the national level (3129.80 cases per 100,000 inhabitants). The northeast Brazil region reported the second-highest number of confirmed deaths from COVID-19; the states of Ceará, Sergipe, Pernambuco, and Paraíba, respectively, had the highest mortality rates in northeastern Brazil. At the national level, the state of Roraima in the northern region showed the highest incidence in the country (10,678.3 cases/100,000 inhabitants), almost tripling the Paraiba index according to the numbers published by the special epidemiological Ministry of Health bulletin [27]. 

The manifestation of the pandemic in Brazil in March 2020 was marked by excess mortality observed in the capitals of the northeast, northern, and southeast regions. However, Paraíba had a higher excess of deaths in the interior compared to the capital [28], suggesting a rapid internalization of the pandemic in this state. The first phase of the pandemic led to collapses in the health system, a care crisis, and high mortality in Intensive Care Units (ICUs); there were hospital overloads, and deaths began to happen outside the ICUs. During this period, Brazil experienced the synchronization of the epidemic curves, which was characterized by the spread of the virus throughout the national territory and by the greater mobility of the population and circulation of SARS-CoV-2 [29].

In 2020, despite the case fatality indicating a decreasing trend with a reduction of 0.40% per day, April had the highest case fatality of the entire period analyzed, with an index of 7.8%. This rate was higher than that described in Brazil (7.0%) and some Latin American countries such as Chile (1.4%), Argentina (5.0%), Colombia (4.5%), and Bolivia (5.3%) [30]. A study in Pernambuco, a state boarding Paraiba, also reported higher case fatality of COVID-19 than in Brazil in April 2020 (8.3%), and the highest index was verified in May of the same year (12.9%) [11]. 

During this period, case fatality varied among regions, and socioeconomic, demographic, political factors, and comorbidities were elements that confirmed those differences [31]. In the Brazilian northeast, there are many differences among the states, mainly social differences; this reflects challenges for health managers and the different repercussions of the pandemic in each region [32].

Even with the advance of the pandemic in the region, the year 2020 showed a decrease in the incidence trend of COVID-19, registering a daily decrease rate of 1.72%. However, it did not indicate that the pandemic was under control, as the low testing capacity for COVID-19 may have contributed to the reduction in the identification of new disease cases. This fact makes it difficult for Epidemiological Surveillance services to track, isolate, and monitor cases of COVID-19. It is necessary to expand and increase diagnostic tests for COVID-19 to accurately recognize the rates achieved by the pandemic [30]. In 2020, Paraíba was among the federation units that least performed COVID-19 tests in the population [33,34]. Overall, during this period, only about 7.8% of cases of COVID-19 in Brazil were reported [35], and there was high variability in the case notification rate between states [36].

### 4.2. 2021: The Year of Massive Vaccination against COVID-19 Started a “Light of Hope”

The scenery of 2021 is characterized by an aggressive second wave of the pandemic, the implementation and progress of vaccination against COVID-19, and the emergence of the Omicrom variant. 

Examining the 2021 data in detail, March to June registered the highest volume of cases and fatalities (Figure 2). These figures reflect the situation at the national level.

An acceleration of the pandemic was visible in Brazil in March 2021, fueled by the spread of new strains of the SARS-CoV-2 virus, such as the P.1 variant found in Manaus, which is associated with cases of reinfection, and the P.2 variant discovered in Rio de Janeiro, related to a more remarkable ability to transmit the disease [37]. Consequently, the result was devastating; on March 23, Brazil registered 3251 deaths in one day for the first time. Then, in less than a month, on April 8, the country sadly recorded a peak in fatalities, reporting 4249 deaths from COVID-19 in 24 h [38]. Most Brazilian states adopted more restrictive measures to mitigate the spread of SARS-CoV-2, only while experiencing a scenario characterized by a high number of deaths and high hospital bed occupancy rates [39]. 

It was a critical phase, marked by collapses in the health system and localized health crises, combined with a lack of equipment, supplies for the ICU, and exhaustion of the health workforce [40].

In the following months, from July to December, a decrease in the number of fatalities was observed. This reduction may be attributed to maintaining non-pharmacological measures and the vaccination program’s high coverage.

Despite the difficulties, 2021 brought a “light of hope” with the worldwide COVID-19 vaccination campaign that started and proceeded massively in 2021 [41]. The World Health Organization (WHO) anticipated that as the volume of vaccinated people increased, the circulation of the virus would decrease, reducing the risk of mutations [42]. However, the imbalance in vaccine distribution [43] impacted the weighty load caused by this pandemic [44]. Nations with fewer resources had more difficulty acquiring the vaccines and immunizing their population; to this, we must add the willingness of the people to receive the vaccine [45]. Such differences influenced the epidemiological control of the pandemic, reducing millions of lives globally [46].

Brazil did not escape this global reality [47]; having a vast geographic extension with deep socioeconomic contrasts, it began the vaccination campaign against COVID-19 on 17 January 2021 without a coordinated national effort [48]. Nevertheless, the solid experience of the federal vaccination program implemented through the Public Health System (SUS) made accelerating the vaccination plan for adults aged +18 and priority groups possible. Advances occurred throughout this first year of vaccination in the country, but not homogeneously; Brazil’s south and southeast regions had a high percentage of the population immunized, while the midwest, north, and northeast needed more immunization for COVID-19 [40].

Paraiba started vaccination against COVID-19 in early 2021, obeying the protocol of the National Immunization Program, with massive participation among adults over 18. It followed the same strategy as the rest of the Brazilian states, prioritizing health workers, the elderly, indigenous communities, and institutional people. The focus was to protect the population from the effects of new variants and minimize the number of victims that progressed to death [49]; this could explain the high percentage of coverage over 18 years. The vaccination coverage in Paraiba reflects the results reached at the national level. By December 2021, Brazil achieved 80% of its target population fully vaccinated [50].

In Paraíba, the year 2021 ended with decreasing trends in the incidence and mortality from COVID-19. However, case fatality showed growth trends at a daily rate of 0.15%, which led the region to experience a new, worrying prospect. 

### 4.3. 2022: The Emergence of the Omicron Variant Significantly Altered the Trajectory of the Ongoing Pandemic

In the period from December 2021 to January 2022, a new wave of transmission was verified in the country, coinciding with Christmas and New Year festivities, vacations, relaxation of mobility restriction measures, and the introduction in the land of the Omicron variant, Influenza A virus epidemic in several municipalities, which led to an increase in cases of Severe Acute Respiratory Syndrome [40]. In addition to these factors, simultaneously, the state of Paraíba also faced syndemic problems, with notifications of cases of Dengue, Chikungunya, and Zika virus [51]. 

After massive adult population vaccination in Paraiba, with a significant positive impact on the overall incidence and mortality rates of COVID-19 in 2021, these indicators rapidly increased by February 2022. Our findings reveal that incidence reached the highest peak of the period, 1841 cases per 100,000 inhabitants, and mortality ran at 9.1 deaths per 100,000 inhabitants. This enlargement in the number of cases and the significant growth in incidence rate at the beginning of 2022 coincides with the Fiocruz observatory report [42], which indicates that the BA variant was responsible for the COVID-19 outbreak that occurred in the country between the end of 2021 and January 2022. The BA.2 variant spread in Brazil and other countries, and the BA.4 and BA.5 strains spread faster than the mutations previous to Omicron. Despite the exponential growth in cases and incidence of COVID-19 in Paraíba in January and February 2022, the number of fatal victims did not grow in the same proportion. 

This deterioration corresponds with the arrival of the Omicron variant, identified in South Africa and Botswana in November 2021, which the World Health Organization (WHO) defined as a variant of concern due to its large number of mutations [23] being more transmissible than the original strain of the virus. According to the Centers for Disease Control and Prevention (CDC) [52], the Omicron variant spreads more quickly than the original SARS-CoV-2 virus and the Delta variant. After one year of the emergence of the Omicron variant, it has been constantly changing. As of November 2022, more than 500 subtypes or variations of this variant are circulating. These different versions of the Omicron variant are quite similar in their impact on public health [53]. Consequently, the strategies and measures needed to address them are similar across the various sublineages.

Vaccines have played a crucial role in mitigating the impact of the Omicron variant; however, research indicates that vaccine effectiveness against various aspects, such as infection, disease, and mortality, decreased gradually over time, albeit at varying rates. Nevertheless, their efficacy in preventing severe outcomes like hospitalization and death has remained consistently high, saving countless lives. 

Overall, the end of 2022 revealed a better picture in Paraiba. On comparing the epidemiological indicators concerning those observed in the previous years, the incidence decreased by 0.41% per day. However, it is necessary to highlight that this may reflect that the population reduced the search for testing. Even with good scenery, the pandemic in Paraíba, like Brazil, is constantly evolving. Additionally, evidence about the long-term effects on people who suffer from COVID-19 has been published. A systematic review by Lopez-Leon, S. et al. [54] registered more than 50 disorders among post-COVID-19 patients, suggesting that those sequelae and the high morbimortality of COVID-19 could decline life expectancy [55].

The spread of the virus and the high registers of cases and deaths compromised the structure of the public and private health system due to the exponential increase in primary health services and the need for more complex services that require hospitalization and invasive ventilation [56]. Before the pandemic, the deficient conditions of health coverage and hospital infrastructure were already visible issues in this territory. This situation worsened significantly due to the morbidity and mortality caused by COVID-19 [10], which reflects Paraiba’s vulnerability, with specific characteristics that determined the course of the pandemic in this state.

### 4.4. The Socioeconomic Context of Paraiba and Its Potential Impact on the COVID-19 Pandemic

The map of the new poverty in Brazil, research conducted by the Getulio Vargas Foundation [57], places Paraiba among the states with the highest poverty rate, with 47.18% of its population with a monthly income of fewer than BRL 497 (less than USD 100). Of the seven states with the highest poverty index, five are from the northeast region (Maranhao, Alagoas, Pernambuco, Sergipe, and Bahia), and one is from the northern region (Amazonas). Maranhão is the state with the highest poverty rate (57.90%).

Being a state with profound socioeconomic contrasts, groups of significant social vulnerability coexist in this territory. In this context, it is essential to mention the presence of indigenous groups dispersed throughout the Brazilian territory.

According to the latest IBGE census, the northeast region shelters 232,739 indigenous people, of which 10.7% are settled in Paraiba. These communities are significantly more vulnerable to epidemics; they live predominantly in remote places, have precarious socioeconomic and health conditions, and have a shortage of human resources in the health area. They also have communication limitations in the native language. Living in collective houses and sharing utensils favor the spread of the virus. It is said that the indigenous population affected by COVID-19 is under-reported [58]. Evidence indicates that indigenous communities have suffered from exogenous illnesses. During the chaos caused by the H1N1 flu in 2009, their mortality rate was 4.5 times higher than the rest of the general population of Brazil [59].

The number of cases, victims, and the corresponding incidence, mortality, and case fatality caused by the coronavirus from 2020 to 2022 in Paraiba suggests that numerous factors influenced the epidemiology of COVID-19 in the state, such as sociodemographic factors, comorbidities [31], limited health infrastructure and resources [31,40], low human development index, vulnerability, poor adherence to non-pharmacological practices [26], new variants [23,37,40,52], and vaccination [3,50]. 

**Figure 4:**
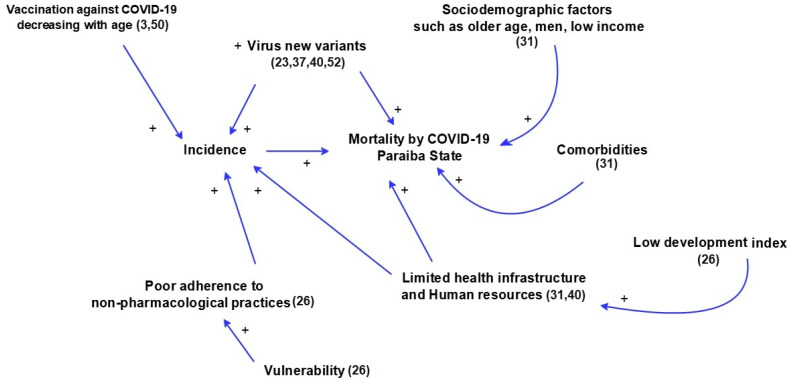
A causal diagram showing the key factors influencing COVID-19 mortality in Paraiba. The vaccination [3,50], new viral variants [23,37,40,52], sociodemographic factors [31], comorbities [31], vulnerability, poor adherence to non-pharmacological practives, low development index [26], and limited heath infrastructure an human resouces [31,40] were the key factors influencing COVID-19 mortality in Paraiba.

Figure 4 depicts a causal diagram that visually represents the key factors influencing COVID-19 mortality in Paraiba, as well as how these factors interact with each other. 

Despite all the efforts made during the last three years of the pandemic, the global response to COVID-19 still needs to be improved and more cohesive, and 2023 requires constant attention [48]. Therefore, the scientific society and Public Health managers must continue to monitor data to develop quick and effective coping strategies.

## 5. Limitations

The data for this study were sourced from official government platforms, but it is important to note that reported cases and deaths may be higher due to delays in reporting. Additionally, the limited availability of widespread COVID-19 testing can result in an undercount of asymptomatic cases, potentially causing a decrease in reported case numbers. Furthermore, the study’s scope was constrained by the lack of detailed sociodemographic information in the governmental database. 

On the positive side, according to the author’s knowledge, this research is pioneering in retrospectively analyzing COVID-19 incidence, mortality, and case fatality rates in Paraiba. It sheds light on this vulnerable region’s dynamics, providing valuable insights despite these limitations. This temporal analysis reveals the specific challenges this region faces, aiming to equip decision-makers with valuable information to respond more effectively to the difficulties experienced by the people living there.

## 6. Conclusions

Over the period from 2020 to 2022, the state of Paraíba in northeastern Brazil experienced significant fluctuations in its COVID-19 epidemiological indicators, including incidence, mortality, and case fatality rates. In 2020, the highest case fatality rate was recorded in April at 7.8%, but it gradually decreased by the end of the year. Despite this decline, the daily growth rates for incidence and mortality remained at 1.72 and 0.42, respectively.

The situation in 2021 was particularly challenging, marked by a surge in COVID-19 deaths, especially in March and April, which saw the highest mortality rates of the entire analyzed period, reaching 30.3 and 25.5 deaths per 100,000 inhabitants, respectively.

However, in 2022, there was a noticeable improvement in the region’s indicators. Case fatality rates decreased, while incidence and mortality rates remained relatively stable. Notably, in February 2022, the state’s mortality rate resembled that of the same month in the previous year, when the population still was without vaccination.

These findings illustrate the dynamic nature of the pandemic, emphasizing the importance of continuously monitoring epidemiological indicators as the situation evolves.

## Figures and Tables

**Table 1:** Municipalities by health region and their proportion by HDI are classified as high, medium, and low.

	Municipalities			
Health Region	Low HDI	Middle HDI	High HDI	Total
R-1	19	4	2	25
R-2	21	4	-	25
R-3	27	13	1	41
R-4	9	3	-	12
R-5	6	11	-	17
R-6	13	9	2	24
R-7	13	5	-	18
R-8	7	3	-	10
R-9	7	8	-	15
R-10	10	5	-	15
R-11	6	1	-	7
R-12	13	1	-	14
Total	151 (67.71%)	67 (30.05%)	5 (2.24%)	223

R: Health Region.

**Table 2:** Prais–Winsten regression estimates and daily percent change of incidence, mortality, and case fatality of COVID-19 in Paraiba state from March 2020 to December 2022.

Period	Incidence			Mortality			Case fatality		
	DPC (IC 95%)	*p*	Trend	DPC (IC 95%)	*p*	Trend	DPC (IC 95%)	*p*	Trend
2020	1.72 (0.96:2.48)	<0.001 *	Increasing	0.42 (0.11:0.74)	0.008 *	Increasing	−0.40 (−0.59:−0.22)	<0.001 *	Decreasing
2021	−0.93 (−1.12:−0.75)	<0.001 *	Decreasing	−0.70 (−0.87:−0.53)	<0.001 *	Decreasing	0.15 (0.02:0.28)	0.023 *	Increasing
2022	−0.41 (−0.71:−0.10)	0.009 *	Decreasing	−0.10 (−0.30:0.11)	0.356	Flat	−0.01 (−0.26:0.24)	0.93	Flat

DPC—daily percent change (%); CI 95%—confidence interval 95%; *p*-value—the probability of statistical significance. The Prais–Winsten regression test detected a statistical difference, * *p* < 0.05.

## Data Availability

Data were extracted from https://covid.saude.gov.br/. Accessed on 23 January 2023.
